# Lessons learned from the introduction of genetically engineered crops: relevance to gene drive deployment in Africa

**DOI:** 10.1007/s11248-022-00300-2

**Published:** 2022-05-11

**Authors:** Hector Quemada

**Affiliations:** Department of Biological Sciences, Western Michigan University, Kalamazoo, MI 49008 USA

**Keywords:** GE crops, Agricultural biotechnology, Gene drives, Lessons learned, Europe, Africa

## Abstract

The application of gene drives to achieve public health goals, such as the suppression of *Anopheles gambiae* populations, or altering their ability to sustain *Plasmodium* spp. infections, has received much attention from researchers. If successful, this genetic tool can contribute greatly to the wellbeing of people in regions severely affected by malaria. However, engineered gene drives are a product of genetic engineering, and the experience to date, gained through the deployment of genetically engineered (GE) crops, is that GE technology has had difficulty receiving public acceptance in Africa, a key region for the deployment of gene drives. The history of GE crop deployment in this region provides good lessons for the deployment of gene drives as well. GE crops have been in commercial production for 24 years, since the planting of the first GE soybean crop in 1996. During this time, regulatory approvals and farmer adoption of these crops has grown rapidly in the Americas, and to a lesser extent in Asia. Their safety has been recognized by numerous scientific organizations. Economic and health benefits have been well documented in the countries that have grown them. However, only one transgenic crop event is being grown in Europe, and only in two countries in that region. Europe has been extremely opposed to GE crops, due in large part to the public view of agriculture that opposes “industrial” farming. This attitude is reflected in a highly precautionary regulatory and policy environment, which has highly influenced how African countries have dealt with GE technology and are likely to be applied to future genetic technologies, including gene drives. Furthermore, a mistrust of government regulatory agencies, the publication of scientific reports claiming adverse effects of GE crops, the involvement of corporations as the first GE crop developers, the lack of identifiable consumer benefit, and low public understanding of the technology further contributed to the lack of acceptance. Coupled with more emotionally impactful messaging to the public by opposition groups and the general tendency of negative messages to be more credible than positive ones, GE crops failed to gain a place in European agriculture, thus influencing African acceptance and government policy. From this experience, the following lessons have been learned that would apply to the deployment of gene drives, in Africa:

It will be important to establish trust in those who are developing the technology, as well as in those who are making regulatory decisions. Engagement of the community, where those who are involved are able to make genuine contributions to the decision-making process, are necessary to achieve that trust. The use of tools to facilitate participatory modeling could be considered in order to enhance current community engagement efforts.

Trusted, accurate information on gene drives should be made available to the general public, journalists, and scientists who are not connected with the field. Those sources of information should also be able to summarize and analyze important scientific results and emerging issues in the field in order to place those developments in the proper context. Engagement should involve more opportunities for participation of stakeholders in conceptualizing, planning, and decision-making.

Diversifying the source of funding for gene drive research and development, particularly by participation of countries and regional bodies, would show that country or regional interests are represented.

Efforts by developers and neutral groups to provide the public and decisionmakers with a more thorough understanding of the benefits and risks of this technology, especially to local communities, would help them reach more informed decisions.

A better understanding of gene drive technology can be fostered by governments, as part of established biosafety policy in several African countries. Developers and neutral groups could also be helpful in increasing public understanding of the technology of genetic engineering, including gene drives.

Effective messaging to balance the messaging of groups opposed to gene drives is needed. These messages should be not only factual but also have emotional and intuitive appeal.

## Introduction

The development of genetically engineered gene drives offers a range of new applications of genetic engineering (GE) that have not been readily achievable with this technology to date, particularly in the genetic engineering of populations. Currently, genetic engineering of populations has been primarily achieved in agricultural settings (crop and livestock) through controlled crossing of individuals with the transgene of interest into desired genetic backgrounds. In GE crops, controlled breeding has been the means by which populations (cultivars and hybrids) for deployment to farmers have been produced. Informal selective breeding could also be the means by which desirable genes could be propagated throughout cultivars maintained by subsistence farmers. However, the ability to increase frequencies of a desired transgene in natural populations has been limited to exploiting the selective advantage that it might confer. This mechanism would be the means by which a disease resistance gene could be deployed in an endangered uncultivated species, such as the American chestnut.^1^ This selective advantage would require many generations to achieve complete penetration of the gene throughout a population and would likely require a transgene with a strong selective advantage. Gene drives offer a more effective process for increasing gene frequencies in populations through a rapid conversion of alleles. Thus, the reliance on natural selection to achieve high frequencies is replaced by mechanisms at the molecular level that confer a reproductive advantage to specific alleles.

The application of gene drives to achieve public health goals, such as the suppression of *Anopheles gambiae* populations or altering their ability to sustain *Plasmodium* spp. infections, has received much attention from researchers. If successful, this genetic tool can contribute much to the wellbeing of people in regions severely affected by malaria. However, gene drives are a product of genetic engineering, and the experience to date, gained through the deployment of GE crops, is that GE technology has been slow to receive public acceptance in Africa, a key region for the deployment of gene drives. It is possible that the experience with GE crops in that region has laid the foundation for future resistance to other GE technologies. Therefore, it is useful to examine that past experience to capture any lessons that might be learned from it that could be used to improve public acceptance of GE mosquitoes with gene drives, thereby improving the chances of regulatory and general government approval by countries in the region.

This report will begin by surveying the status and history of GE crop deployment throughout the world, to provide the background context for this subject. This history of GE crops provides good analogs for gene drive development and deployment, since very few GE organisms have been deployed on the same scale in the environment (albeit in managed rather than wild systems). Nevertheless, the amount of area over which GE crops have been deployed is on a scale like that which is anticipated for some gene drive applications. Other GE organisms that have been deployed for different fields of use, such as GE salmon and GE non-driving mosquitoes for control of arbovirus disease will not be covered directly, since the experience with these applications has not been as extensive. However, the issues pertaining to these cases largely overlap with those of GE crops.

While not dealt with in detail, the experience with non-driving GE mosquitoes will be briefly mentioned here. In those cases where non-driving GE mosquitoes engineered to control *Aedes aegypti* populations for prevention of arboviral diseases have been tested and released, acceptance has been mixed. Large scale release and field testing have been readily allowed and accepted in Brazil, Panama and the Cayman Islands, but resisted in the United States (Berube 2020). In the case of GE *Anopheles gambiae*, contained experiments and one test of GE male sterile mosquitoes have already received criticism from groups generally opposed to GE technology (Cisnetto and Barlow 2020). While these tests and releases of non-driving GE mosquitoes have been allowed by some regulatory jurisdictions, international regulatory policy has not yet coalesced into a standard set of regulatory requirements applicable to gene drives. The scientific achievements in this field are rapid and will present a challenge for regulatory agencies to keep pace, much in the same way that GE crops challenged regulatory systems in the past, especially in developing countries.

In this report, GE organisms are those that have genes inserted through recombinant DNA techniques. While organisms produced through gene editing might also be classified as GE in some jurisdictions, the experience with these organisms is still accumulating, and their status in the public view as GE organisms is not yet clear. Therefore, information from experiences with gene edited organisms will not be covered.

## GE crop adoption, their benefits, and their safety

### Adoption of GE crops worldwide

**Fig. 1:**
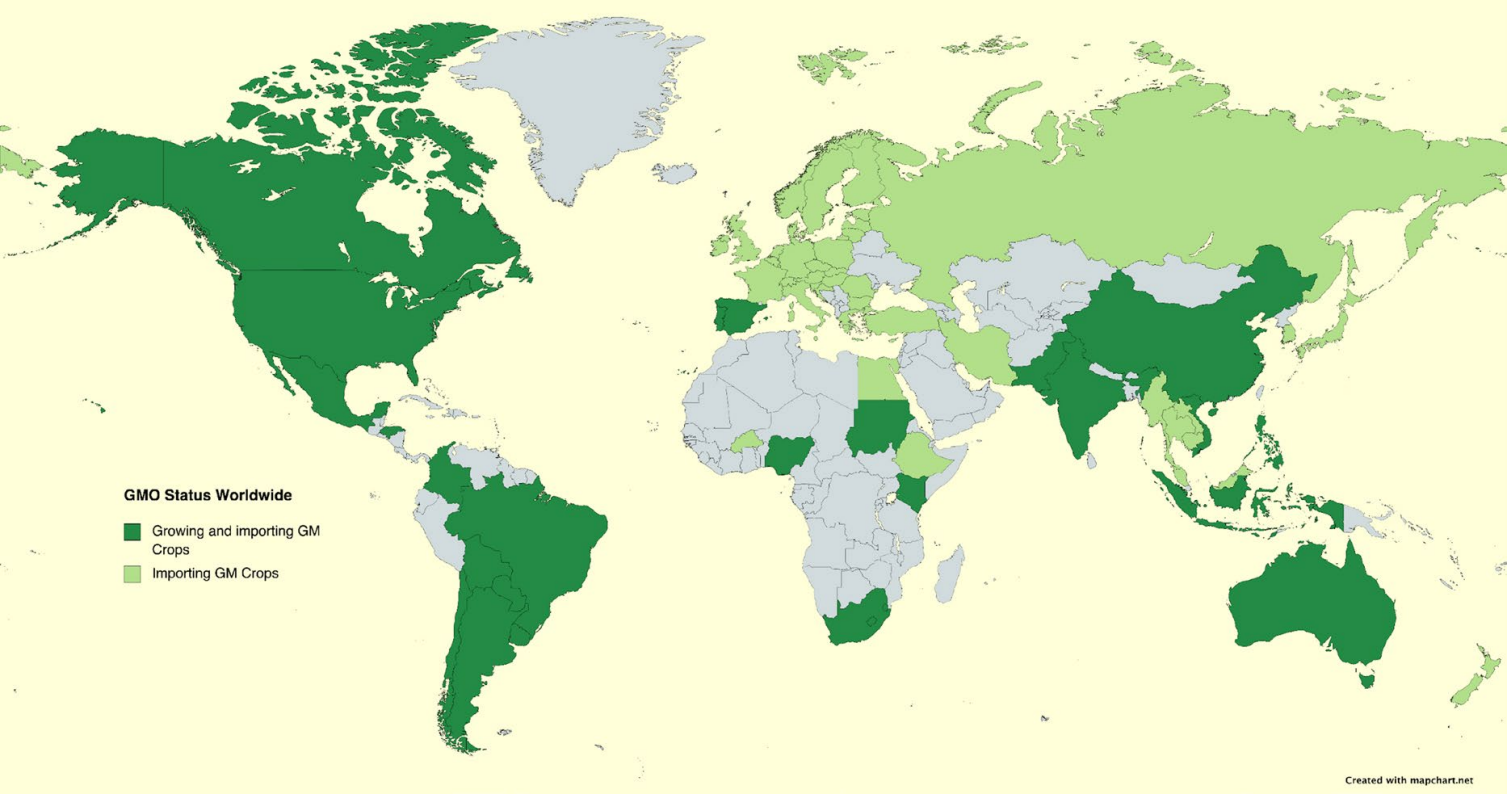
Status of GE crop commercial deployment worldwide. *Dark green* countries growing and importing GE crops; *light green* countries importing GE crops. Source: (ISAAA 2018)

Genetically engineered crops have received regulatory approvals and have been in commercial production for 26 years, since the planting of the first GE soybean crop in 1996. As of 2018, 26 countries grew 192 million hectares of GE crops, with the United States, Brazil, Argentina, Canada, and India accounting for the vast majority of that area. In addition to these countries, 44 are importing GE crops (Fig. 1). In Argentina, 18 million hectares of GE soybeans, 5.5 million hectares of GE maize, and 0.37 million hectares of cotton were planted in 2018, at an adoption rate (relative to total crop area) of 99% for GE herbicide tolerant soybeans, 97% for GE insect resistant and herbicide tolerant maize, and 93% for insect resistant GE cotton (ISAAA 2018). In the United States, GE insect resistant and herbicide tolerant maize accounted for 92%, GE herbicide tolerant soybeans 94%, and GE insect resistant cotton 96% respectively, of the acreage planted in 2020^2^^,^ 3. A striking feature of the map shown in Fig. 1 is the relative absence of European and African countries growing GE crops, or in the case of Africa, even importing them. One possible explanation for this phenomenon will be explored in this report and will also serve as the focus of the lessons learned from this field that could be applied to the research and development of gene drives.

### Benefits of adoption of these crops

The high level of adoption throughout the world has resulted in significant socio-economic, health and environmental benefits. In 2018, farm income worldwide from GE crops was US$18.95 billion, and the cumulative total income since 1996 was US$ 225 billion, with half of that income earned by developing country farmers (Brookes and Barfoot 2020a). In fact, yield and profitability has proven to be higher in developing countries than in developed countries (Carpenter 2010; Klümper and Quaim 2014). The GE crops in production today fall into two main categories—those expressing insect resistance and those expressing herbicide tolerance. These two categories have been the most extensively studied regarding their impacts and benefits.

#### Benefits of insect resistant crops

Since 1996, the use of insect resistant GE crops expressing various insecticidal proteins from the bacterium, *Bacillus thuringiensis* (Bt), has resulted in a 775.4 million kg reduction in pesticide use (Brookes and Barfoot 2020b), and as a result, decreased the environmental impact associated with herbicide and insecticide used on these crops. They have also directly and indirectly reduced the exposure of farmers who did not use them (Shelton et al. 2002; Cattaneo et al. 2006; Hutchison et al. 2010; Kathage and Qaim 2012; Perry et al. 2016; Dively et al. 2018) (Brookes and Barfoot 2020b). The reduced exposure presumably has long-term health benefits, but an immediate effect has also been experienced in the reduction of farmer pesticide poisonings, as has been reported for China, India, Pakistan and South Africa (Smyth 2020; and references therein, and Kouser et al. 2019). For insect resistant maize, human health benefits are also expected due to the reduction in levels of mycotoxins, which are toxic—causing neural tube defects—and carcinogenic (Smyth 2020). These effects are particularly beneficial in those developing countries where maize is the staple food (for example, Honduras, Macall et al. 2020). Environmental benefits are expected from the reduction in chemical pesticide use, through the known impacts of these pesticides on wildlife (Siegrist and Hartmann 2020; Brookes and Barfoot 2020b; Brookes and Dinh 2020; Ahmed et al. 2020). One expected impact of the reduction in chemical pesticide use is the increase in biodiversity, which has been observed in numerous studies and summarized by Carpenter (2011). Furthermore, there is evidence that the use of insecticide resistant crops not only reduces the amount of pesticide applied to that crop but also contributes to the reduction of pesticide applied generally (Wu et al. 2008).

Increased yield and economic benefits of insect-resistant GE crops have been well-documented for developed countries, such as the United States (Fernandez-Cornejo and Caswell 2006; Fernandez-Cornejo et al. 2014). However, studies reporting the same benefits in developing countries have also been documented, including Bangladesh (Shelton et al. 2020; Ahmed et al. 2020), India (Kathage and Qaim 2012), Pakistan (Kouser and Qaim 2014a, b), Honduras (Macall et al. 2020), South Africa (Ismael et al. 2002; Gouse et al. 2004; Hofs et al. 2006; Shew et al. 2021) and Burkina Faso (Hema et al. 2017; Bourgou et al. 2020).

Even in Europe, where only Spain and Portugal have had significant experience with only one GE crop event (Sect. “Regulatory status in Europe” below) economic and environmental benefits have been realized. Brookes (2019) reported that in the 21 years since maize was first cultivated on the Iberian Peninsula, a total of 1.65 million hectares have been planted, with farmers benefiting from an increase in income of €285.4 million, primarily due to higher crop yields. Insecticide spraying has also decreased by 678,000 kg of active ingredient, with inferred decreases in environmental impact and health. Kovak et al. (2021) calculate that cultivation of GE crops in Europe (soybean, maize, cotton, canola, and sugarbeet) could have reduced the region’s greenhouse gas emissions by 33 million metric tons of CO_2_ equivalents per year, or 7.5% of total EU agricultural greenhouse gas emissions in 2017.

An interesting recent development has been the successful use of Bt cotton in the United States, in concert with classical pest control methods to eradicate pink bollworm, a major pest of cotton (Tabashnik et al. 2021). This example illustrates the compatibility of GE crops in an integrated pest management strategy to solve long-standing agricultural problems.

#### Benefits of herbicide tolerant crops

Crops genetically engineered to be tolerant to herbicides are the other major category. In contrast to insect-resistant crops, yield benefits have not been consistently observed (Fernandez-Cornejo and Caswell 2006; Carpenter 2010; Bonny 2011; Fernandez-Cornejo et al. 2014; Klümper and Quaim 2014). The available data indicate that the yield advantage provided by herbicide tolerant crops are greater for developing country farmers than for their counterparts in developed countries (Carpenter 2010). Despite the mixed reports of yield advantages of herbicide tolerant crops, their continuing increased adoption indicates that benefits are derived from these crops other than yield alone. The reduced management cost of weed control made possible by these crops tips the balance sheet toward greater overall farm profitability (Green 2012). Easier and more flexible weed control results in reduced on-farm management time (Sankula et al. 2005; Fernandez-Cornejo 2006; Desquilbet et al. 2019). Freeing up time provides consequent benefits such as the opportunity for off-farm employment for farmers and spouses, leading to overall greater family income (Fernandez-Cornejo and Caswell 2006).

Herbicide tolerant crops also show economic advantages in Latin America and the United States when used in combination with other traits such as insect resistance (Committee on Genetically Engineered Crops 2016; Brookes 2020; Macall et al. 2020). However, this advantage of combined traits appears to be less straightforward in developing countries (Afidchao et al. 2014). In Eastern Europe and the United States herbicide tolerance provides economic advantages when deployed in higher-yielding varieties (Brookes 2020).

Environmental benefits of herbicide tolerant crops are primarily observed in two areas: (1) soil preservation, reduced fuel consumption, and reduced greenhouse gas emissions through conservation tillage practices, and (2) reduction in the environmental toxicity of the herbicides used in conjunction with those crops. Conservation tillage seeks ways to reduce the frequency or intensity of tillage, in order to preserve soil quality, reduce greenhouse gas emissions, and use of farm machinery (resulting in lower fuel usage) (UC Sustainable Agriculture Research and Education Program 2017). Herbicide tolerant crops have become an important tool in this approach, enabling the increase in no-till acreage from 45 million hectares to 111 million hectares in the decade from 1999 to 2009 (Derpsch et al. 2010). This increase in no-till practices has meant a reduction in soil erosion and increased water conservation (Trigo 2011; Blanco-Canqui and Wortmann 2020). Fuel consumption has also been greatly reduced, totaling 920 million liters, or the equivalent of 1.6 million cars removed from the road in 2018 alone (Brookes and Barfoot 2020b). Because the area planted with GE herbicide tolerant crops has increased, the amount of herbicide applied to crops has increased. However, herbicide use has shifted to less toxic active ingredients, in particular glyphosate, resulting in a reduction in environmentally adverse effects of herbicide use (Brookes and Barfoot 2020b).

### Position of scientific societies and other organizations on the safety of GE crops

The safety of consuming foods derived from approved GE crops has been reviewed numerous times by a wide range of scientific bodies. These include the Society of Toxicology; the US National Academies of Sciences, Engineering, and Medicine; the Council on Agricultural Science and Technology; the American Association for the Advancement of Science; the World Health Organization; The Royal Society; and the American Medical Association (Hollingworth et al. 2003; Phipps et al. 2006; American Dietetic Association 2006; American Medical Association 2012; Board of Directors of AAAS 2012; World Health Organization 2014; Committee on Genetically Engineered Crops 2016; The Royal Society 2016). These organizations unanimously agree that food derived from approved GE crops is safe. Broader political support has also been expressed by a wide range of scientists. For example, several past winners of the Nobel Prize have expressed their support for agricultural biotechnology in an open letter addressed to opposition groups, governments, and the United Nations.^4^ Academic institutions with significant investment in agricultural research and development have also provided much information online to support the conclusion of safety of foods derived from GE crops.^5^

## Opposition to GE crops in Europe and its impact on Africa

### Continuing opposition

Despite the documented benefits to farmer income, health and the environment, and the high degree of agreement about the safety of GE crops that have been approved for public use, opposition to GE crops among consumers remains strong in many parts of the world, especially Europe^6^ (Zilberman et al. 2013; ISAAA 2018), with its consequent influence on Africa (Huffman et al. 2003; Gheysen et al. 2019). Despite the large body of scientific evidence, some European scientists continue to strongly question the food and environmental safety of these crops (Hilbeck et al. 2015, 2020).

Europe retains a precautionary stance to newer technologies, such as gene edited crops (van der Meer et al. 2020). In the case of France, this has even extended to the capturing of new herbicide-tolerant varieties created by random mutagenesis as GE^7^, even though such plants have historically been considered products of traditional breeding.

### Regulatory status in Europe

Regulatory restrictions on GE crops in Europe is especially evident in the lack of authorization to cultivate these crops. Europe’s regulatory system grants authorization for growing GE at the European Union level,^8^ but individual member states can prohibit growing them in their jurisdiction. Authorization to grow GE crops has been difficult to obtain in Europe, with only eleven products (“events”^9^) having received such authorization: seven carnation, one cotton, two maize, and one potato.^10^

However, only one event, an insect-resistant maize (event MON810), is currently being grown in the EU, and only in two member states, Spain and Portugal. Spain has been cultivating this event since 1998, and Portugal since 2012 (Fernandes et al. 2014; Brookes 2019; Brookes and Barfoot 2020b). In the past, MON810 was grown in as many as four of the EU member states during 2015 and 2016 but by 2017 only Spain and Portugal continued this cultivation (Rostoks et al. 2019). Some have argued that the restrictions on GE crops has had a negative impact on European agriculture (Stanciu and Sarbu 2019)^11^, ^12^.

Opposition to the authorization of these crops continues in the European Parliament, which as recently as November 4, 2020, adopted a resolution calling on the European Commission not to authorize placing on the market any GE plants containing genes that confer antibiotic resistance, which are often used as a selectable marker in the transformation process^13^. This prohibition would effectively stop all imports of genetically engineered soybeans and maize—commodities that are key components of animal feed—into the EU (European Parliament 2020). This recent resolution highlights the ongoing tension between the political and scientific considerations surrounding the approval of GE organisms in Europe, due to the legally inherent involvement of both components in the decision-making process^14^.

A major source of opposition to GE crops in Europe is a view of agriculture that differs from countries such as the United States, where production of GE crops is highest (Zilberman et al. 2013; Lucht 2015). The latter emphasizes higher reliance on intensification and technology, while the former is more resistant to its use (Pratt 2020). The increasing influence of the agroecology^15^ movement exemplifies this view. Agroecology and organic farming hold a key position in the European Biodiversity Strategy (European Commission 2020a). The competition from cheaper agricultural produce from countries such as the United States, produced by more intensive methods, therefore threatens the European vision for agriculture, and underlies much of the opposition to GE crops (Juma 2016).

Europe’s approach to agriculture is primarily contained in its Common Agricultural Policy (CAP), which since 2010 has increasingly included elements that are designed to address environmental and social challenges in addition to production and market concerns. This direction appears to align well with the wishes of the European public, despite the conflicting goals of agricultural productivity and environmental concerns represented by different committees contributing the formulation of that policy (Pe’er et al. 2019). A major feature of the CAP, direct payments to farmers, amounted to almost 70% of the budget for implementing the policy in 2017 (Pe’er et al. 2019). Consequently, it can be argued that the European agricultural policy does not provide strong incentives for efficient agricultural production. It is therefore no surprise that the CAP has played an important role in encouraging organic agriculture among its member states^16^. Since organic standards prohibit the use of GE crops in their production systems, the organic agriculture section stands strongly opposed to the introduction of these crops.

The CAP is influenced by other policy initiatives, including the Biodiversity Strategy mentioned above, as well as the Farm to Fork strategy for agriculture (European Commission 2020b), part of the European Green Deal^17^. These policies give further prominence to environmental concerns in European production agriculture relative to agricultural policies in other parts of the world. The strategy also requires that places where food is sourced also adhere to European standards, forcing suppliers in Africa, for example, to adopt the same policies in order to do business with European markets.

### The effect of EU policies on African regulatory decisions

African countries are especially subject to the policies implemented in Europe, due to the size of the market that the latter represents. Consequently, decisions have been made by African governments that might seem detrimental to the well-being of their people but were probably shaped at least in part by the economic power of the European market, which has a strong preference for non-GE foods. An example of such a decision is provided below. This preference is held by a food-secure public that can afford to pay for it (Huffman et al. 2003). In a recent book, South African molecular biologist Jennifer Thomson attributed the high degree of European influence on African policies to its historical colonial involvement with Africa, and continuing aid, trade and educational involvement with the continent (Thomson 2021). Indeed, Africa and the European Union have joint strategic objectives that reflect the shared “rich history with EU countries, but also common values and interests” (European Commission 2019). Thomson’s analysis supports earlier work by Paarlberg, who argued that developed country attitudes were preventing African countries from receiving the benefits of this technology (Paarlberg 2009).

An illustration of this influence on African policies can be seen in certain countries’ response to food aid two decades ago, with continuing effects today. In 2002, Southern Africa (Zambia, Mozambique, Malawi, Zimbabwe, Swaziland and Lesotho) faced a food crisis (Huffman et al. 2003; Dorward and Kydd 2004; Zerbe 2004). More than 15 million people faced imminent hunger while 3 million faced starvation due to several factors, including climate (leading to drought), HIV/AIDS, structural adjustment, debt, collapsing public services, and poor governance (Huffman et al. 2003; Zerbe 2004). Despite this crisis, these six nations rejected food aid in the form of maize (a staple crop in that region) because the maize, which came from the United States, was genetically engineered. This decision was taken despite assurances of safety by the United Nations World Food Programme, which was responsible for distributing the aid^18^^,^ 19. Juma (2016) attributed this decision in part to diplomatic pressure from the EU, leveraged by their trade ties. More specifically, Huffman et al. (2003) ascribed the refusal of the transgenic maize to the fear that Europe would ban African agricultural exports if they became contaminated with GE maize components. On the other hand, some critics of the food aid (Zerbe 2004) argued that it was the United States that was exploiting this situation as a means of promoting biotechnology in Southern Africa, thus advancing the dependence on US-based multinational companies, weakening local production capacity and ultimately making food insecurity worse. Activist groups also charged the United States with using the donations to dump unsold maize^20^. There was also a successful campaign by non-governmental organizations such as Greenpeace to characterize the maize as “Frankenfoods” (Huffman et al. 2003). This refusal of GE maize has continued, with Zimbabwe only recently easing its position, once again because of impending famine^21^^,^ 22.

This slow adoption of GE crops in Africa (Fig. 1), has had an economic cost as well, in terms of foregone benefits. The cost of delay in realizing the benefits of this technology has been calculated by Wesseler et al. (2017). These authors analyzed three crops—cowpea, maize, and banana (matooke)—with respect to economic and nutritional benefit (translated to economic terms), in Benin, Niger, Nigeria, Kenya, and Uganda. A one-year delay in the deployment of cowpea in the three West African countries cost US$64 million combined foregone economic benefit, and US$50 million to US$97 million for a one-year delay in deployment of maize and matooke in Kenya and Uganda, respectively.

### Movement by African countries to develop more GE-friendly agricultural policies

However, there are indications that some African countries are now realizing the need to develop genetically engineered crops through their own efforts, to suit their own needs. Disease resistant bananas, insect resistant cowpea, virus resistant cassava and maize, drought tolerant maize, and late blight resistant potato are currently in development on the continent, to meet challenges that African farmers are experiencing in crops that are consumed by Africans (Thomson 2021). In contrast to the corporate funding that has driven most GE crops to date, these projects have been developed with funding from governments or private donors, with the participation of scientists at national government-supported research institutions. Likewise, regulatory instruments are being established that achieve the national goals of African countries. For example, Kenya, Nigeria, and Eswatini are developing policies that would allow them to evaluate and regulate gene edited and gene drive organisms, and not waiting for developments in Europe or elsewhere to dictate those policies (Meeme 2021). Kenya is also enacting reforms in its agricultural sector that would encourage the use of Bt cotton to strengthen the country’s position in fiber crops.^23^

### Developing opposition to gene drives in Europe

Consistent with attitudes toward GE crops, some advocacy groups report that public attitudes in Europe currently are overwhelmingly critical of gene drives (Duboua-Lorsch 2021), with 70% opposed to the release of these organisms into the environment, and that any release should be postponed until they are proven harmless to biodiversity, health, agriculture, or peace^24^. Activism in Europe is organizing against this technology^25^. Activist groups have been successful in convincing the European Parliament to call for a global moratorium on the release of gene drives, including experimental releases, at the upcoming Conference of the Parties of the Convention on Biological Diversity (as of this writing, planned for the latter half of 2022)^26^^,^ 27.

### Movement by African countries to develop more GE-friendly public health policies

Paralleling the apparent African shift in attitudes to genetically engineered crops, there is also a trend toward policies that allow a more favorable view of GE technology in the area of public health, particularly gene drive applications. For example, the African Union High Level Panel on Emerging Technologies urged in a report that African member states should support laboratory, field and semi-field studies to evaluate the potential of gene drives to contribute to the elimination of malaria in Africa, and support research that would lead to optimization of this technology (African Union and NEPAD 2018). This report received political strength through its endorsement by the African Union Executive Council^28^. Consistent with the report, the African Union Development Agency-New Partnership for Africa’s Development (AUDA-NEPAD) established the West Africa Integrated Vector Management platform (WAIVM), which has been developing guidelines for African countries to follow when establishing regulations for research and development of integrated vector management tools, including gene drives^29^.

The malaria burden in Europe, where there have been no reported indigenous cases since 2014 and no indigenous deaths from 2000 to 2019 (World Health Organization 2020), is minimal compared to Africa. With this in mind, African scientists are also becoming more willing to address the inconsistency between European policy objectives and needs of their own continent. For example, one recent op-ed article by a Tanzanian malaria researcher^30^ points out that.

“Decisions made in Europe have ramifications for Africa and beyond, and by clearing the path for innovation, the European Parliament can set a precedent for supporting scientists. They can choose science over fear; and a shared responsibility over narrow ideology.”

— Fredros Okumu, Ifakara Health Institute, Tanzania

## Lessons learned

Given the documented benefits that have been attributed to GE crops, one would expect that adoption of this technology by the public would be rapid. Likewise, one would expect that gene drive technology as a tool for eliminating malaria would be readily adopted as well. However, this expectation is often not realized for new technologies or solutions. Everett Rogers, developer of a theoretical framework called “Diffusion of Innovation Theory” reminds us that.

“Many technologists believe that advantageous innovations will sell themselves, that the obvious benefits of a new idea will be widely realized by potential adopters, and that the innovation will diffuse rapidly. Seldom is this the case.”

— Rogers (2003)

This section therefore focuses on examining the factors leading to the lack of public acceptance of GE crops in Europe, since acceptance in that region affects its policies and consequently Africa. Because of the impact of Europe on African policies on GE technologies in general, European attitudes could also affect the acceptance of new advances in the field that hold promise for addressing some of Africa’s most intractable health problems.

### Mistrust of government regulatory agencies

In the 1980’s and 1990’s, at the time when GE crops were being tested and eventually deployed on a commercial scale, major failures of the regulatory system of several developed countries, in particular in Europe, served to erode the public trust in regulatory decisions and the ability of government regulators to assure safety. The first of these was the contamination, with Human Immunodeficiency Virus, of blood used for transfusions in many countries, including the United Kingdom, France, Germany, Switzerland, Denmark, Japan, Canada, and the United States (Weinberg et al. 2002). Investigations into these cases resulted in criminal convictions for several officials in Western Europe and Japan.

Then in 1996, at the time when GE crops were first grown in the field in the United States, thus presenting the possibility that genetically engineered components could also be part of the food supply in Europe, the Secretary of State for Health in the United Kingdom warned of a possible link between Bovine Spongiform Encephalitis (BSE) and a new strain of Creutzfeldt-Jakob disease^31^. Despite reassurances from the Secretary that “the risk from eating beef is now likely to be extremely small”, he did propose some precautionary measures. While objectively, the risk appeared to be low, media and public perceptions of risk were much higher. The lack of a timely government response, and mishandling of communication, led to a mistrust of the reassurances of scientists and the widespread concern about the safety of beef in the country (Lanska 1998; O’Brien 2000). While the role of government in the case of GE crops differed from its role in the BSE case, it can be argued that the mistrust of any government decisions on the safety of a controversial product, such as GE foods, had an impact on the public view of the acceptability of that product.

Therefore, public faith in the assurances of safety from regulatory officials was already low when the first GE product, a canned tomato purée produced by the British multinational company, Zeneca (now part of Astra-Zeneca), went on the market in 1996. Reception was mixed. The UK supermarket chains Sainsbury and Safeway sold the product, but a competitor, Tesco announced that it would not be offering it (BBC Home 1996; Bruening and Lyons 2000). The product remained on supermarket shelves for a few years, with sales of 1.8 million cans clearly labeled as produced from genetically engineered tomatoes (Bruening and Lyons 2000). However, that product eventually disappeared.

While the incidents described above might account for the general public mistrust in government in Europe, that mistrust also existed in the United States, despite that country’s leadership in the commercial deployment of genetically engineered crops, and with no particular triggering events. In the United States in 1987, the United States Office of Technology Assessment conducted a survey that asked the question, “Suppose a Federal agency reported that the use of a genetically altered organism did not pose a significant risk to your community but a national environmental group said it did pose a significant risk. Would you tend to believe the Federal agency or the national environmental group?” Sixty-three percent chose to trust the environmental group over the government agency, while only 26% chose the latter over the former (US Congress, OTA 1987).

Mistrust of governments’ ability to properly regulate gene drives is also an area of concern for developers of this technology. In a survey of Nigerian scientists to gauge their attitudes toward the release of genetically engineered mosquitoes in Africa, the Nigerian government was the least trusted to assess the safety of these organisms (Okorie et al. 2014). This survey was taken prior to the publication of the proof-of-concept work on gene drives, and much information about gene drives has become known both in the scientific literature and the popular press since then. It would be interesting to find out if these attitudes have persisted in the intervening years^32^.

Therefore, it is critical that there is a high level of confidence in the regulatory system of countries that make the decisions on the deployment of a gene drive intervention. This goal will be difficult to achieve with gene drives. As the survey mentioned above indicates, there could be doubt among scientists regarding the ability of the government, at least in Nigeria, to properly regulate gene drive technology. It should be noted that Nigeria has one of the best-established regulatory systems for GE organisms in Africa^33^. If there is doubt among scientists about the capabilities of that country’s regulatory system, the capability of other countries on the continent could be even more in question by this group or the general public. It is possible that the opinions of scientists in Nigeria could have changed with the coming into force of the Nigerian National Biosafety Act in 2015, the year following the survey conducted above, and the consequent establishment of the Nigerian National Biosafety Management Agency. While there are African countries that could be viewed as well-equipped to handle technologies such as gene drives (i.e. Kenya, South Africa, Ghana, and Burkina Faso in addition to Nigeria), there are other countries that are less experienced, and may not inspire confidence in regulatory decisions. Groups opposing the deployment of GE organisms in Africa, including gene drives, often highlight this lack of experience. A group opposed to GE crops has stated,

“African nations lack the expertise, equipment, infrastructure, legislation and regulatory systems to implement effective biosafety measures for GE crops. They also lack the funds to build these up and will therefore have to look for outside funding, which will increase their already heavy foreign debt loads. Should the development of GE agriculture really be a priority for African governments at this point in time?”

-Zachary Mukanya, GRAIN^34^

An additional objection raised by opponents has been the susceptibility of African governments to the pressure exerted by industry and private donors to adopt the technology:

“The picture on GE cultivation bans across Africa is not clear due to the current pressure being put on many African governments by the Biotech industry and the Gates Foundation to lift long-standing bans on the import of unmilled GMO^35^ seeds or unmilled GMO food aid…”

-Sustainable Pulse^36^

This implied weakness of African governments undermines the trust in those governments’ decsions.

The apparent novelty of gene drives also places doubt on the adequacy of governance mechanisms for this technology, especially for national approaches. Challenges to proper governance of this technology has been acknowledged by governments and research groups (National Academies of Sciences, Engineering, and Medicine 2016; Adelman et al. 2017; Delborne et al. 2018), and has highlighted the need for a regional approach to regulation (Marshall 2010; Brown 2017; James et al. 2018; Warmbrod et al. 2020; Kelsey et al. 2020). African scientists have also indicated their concern regarding the ability of existing systems to govern gene drives (Hartley 2021). These concerns arise because gene drives have been contrasted to earlier types of GE organisms in agriculture. The latter pose primarily local safety issues, while risks associated with the former have been speculated to be larger in scale, more extensive in potential ecological impacts, and more likely to trigger social issues (Reynolds 2020).

An important prerequisite for trust in the regulatory system is the existence of an established legal pathway in each country where interventions will be deployed, to allow the steps along the research and development pathway to proceed. A prominent example of this requirement is the long history of efforts to pass a biosafety law in Uganda. Since 2013, the country has struggled to pass this law, in order to make possible the progress of GE crop deployment beyond the field trial stage. Activities from laboratory through field trials are considered research and therefore conducted under the authority of the Uganda National Council for Science and Technology, through the National Biosafety Committee, which is housed within the Council^37^. However, further development of GE crops goes beyond the authority of this council and cannot proceed without a law in place. The country has so far been unable to bring such a law into force, despite being brought twice to the point of Presidential signature. The inability to obtain the President’s signature has been blamed on pressure from groups opposing GE crops, who claimed they were harmful and did not benefit local farmers (Thomson 2021). This inability to establish a law is in part responsible for Ugandan scientist concerns about the ability of the country to properly assess and regulate gene drives (Hartley 2021). Some of this concern might be alleviated by more recent developments by the Ugandan National Environmental Management Agency, which sees a pathway to regulating deployment and commercialization through the country’s Environmental Management Act^38^ (Government of Uganda 2019).

Finally, trust in regulators and scientists is built through an engagement process that gives stakeholders meaningful opportunities to contribute to policies that affect them. This is an area that has been noted as critical in the development of gene drive technology (National Academies of Sciences, Engineering, and Medicine 2016; Kolopack and Lavery 2017; James et al. 2018; Resnik 2018). It has received much attention from many different groups that are associated with gene drives. Efforts at public consultations, particularly in Africa, have been conducted in four regions by the African Union Development Agency-New Partnership for Africa’s Development (Glover et al. 2018; Teem et al. 2019), and with stakeholder groups by technology developers and other researchers (Marshall et al. 2010; Finda et al. 2020; MacDonald et al. 2020; Hartley 2021; Thizy et al. 2021; de Graeff et al. 2021).

Therefore, it will be important to establish trust in those who are developing the technology, as well as in those who are making regulatory decisions. Engagement of the community, where those who are involved can make genuine contributions to the decision-making process, are necessary for gene drive technology to be accepted. The community engagement efforts mentioned above are designed to understand what community attitudes are, thus providing stakeholders an opportunity for their opinions to be heard and for gene drive developers to determine ways to communicate more effectively with them. However, more active engagement in planning and decision-making could improve the level of trust from communities as well. This type of engagement can be achieved through a process called “participatory modeling”. In this process, complex problems involving social and environmental impacts are solved in a collaborative manner, using various modeling and visualization tools, and involving expert modelers, scientists, community members, and public officials. These groups participate in the building of a model to conceptualize a problem, interpret the results of the model, and use it to support decision-making (Zellner et al. 2012; Gray et al. 2018; Voinov et al. 2018; Aminpour et al. 2020; Hedelin et al. 2021).

### Scientific publications claiming adverse effects of GEOs

The mistrust of regulatory agencies and the science behind genetic engineering was exacerbated by key scientific publications that contributed to the view that GE crops were harmful to human health or the environment.

#### A paper by Losey et al. (1999) that raised the possibility that maize expressing *Bacillus thuringiensis* (Bt) proteins were harming populations of monarch butterflies

A few years after the first Bt maize products were approved and introduced, a laboratory study (Losey et al. 1999) reported that monarch butterfly (*Danaus plexippus*) larval survival after four days of feeding on milkweed leaves dusted with pollen from Bt maize was almost half of that observed for larvae fed on control (non-transgenic and non-isogenic) maize leaves. This paper was widely publicized and resulted in a joint effort between industry and US regulatory agencies to conduct research to determine if the laboratory results reflected harm to monarch butterfly populations in the field. It should be noted that since the Bt proteins were intended to kill lepidopteran pests of maize, the impact on other Lepidoptera was expected. In the original risk assessment, regulatory agencies, such as the US Environmental Protection Agency, were mainly concerned with nontarget effects on *endangered* lepidopteran species. One identified species, the Karner Blue butterfly was considered in the risk assessment but judged to be at low risk for exposure due to the butterfly’s host plant. Risk to monarchs was also assumed to be low because of expected exposure of monarchs to the corn pollen and the relatively lower risk relative to Bt spray products, to which the monarchs would be more exposed (Pew Initiative on Food and Biotechnology 2004).

A two year study showed that the risk to monarch butterflies from Bt maize was “negligible”, with only one transgenic event providing cause for concern, due to the expression of the Bt protein in the pollen—the only event to have that pattern of expression. (Hellmich et al. 2001; Oberhauser et al. 2001; Pleasants et al. 2001; Sears et al. 2001; Stanley-Horn et al. 2001; Zangerl et al. 2001). Furthermore, that specific event was removed from the market by its developer, thereby removing the major source of risk to the monarch butterfly (Pew Initiative on Food and Biotechnology 2004). In 2001, the US Environmental Protection Agency, after a standard reassessment of the registration of the Bt crops on the market, renewed their registration of all events (Mendelsohn et al. 2003). While research was able to demonstrate low risk, the perception of harm to monarch butterflies persisted. For example, 10 years later, in a posting dated March 21, 2012, the magazine *Mother Jones* reported that “GM Crops Are Killing Monarch Butterflies, After All”^39^. In an August 31, 2012 post, the website *GMO Evidence* perpetuated the conclusion that “Bt Corn Harms Monarch Butterfly Larvae”^40^.

#### A publication in The Lancet by Ewen and Pusztai (1999) that reported laboratory studies, claiming that transgenic potatoes were harmful to the gastrointestinal tract of rats used in the study

The authors reported that in rats fed with a transgenic potatoes genetically engineered to express a lectin protein for nematode and insect resistance, the gastrointestinal tracts of the test animals showed concerning abnormalities. Findings were first made public in a television interview a year in advance of publication, announcing that potatoes expressing these genes could stunt rats' growth and impair their immune system. When finally published, the study again attracted much attention and criticism in the scientific community^41^ and the media^42^, not only of the researchers’ conduct and flawed research but also of the journal’s procedures (Enserink 1999). It should be noted that the potatoes in question were never submitted for approval, and therefore were never part of the human food-chain. Given the standard toxicity tests that are recommended by Codex Alimentarius, using pure protein rather than the potato used as the test substance (Codex Alimentarius Commission 2003), the effects of the transgene-expressed protein would have been detected in any case, and would have led to the rejection of an application for approval, or more likely, the withdrawal of the product during the development process, prior to a regulatory application. Despite the fact that this potato was never consumed as food, the study contributed to the perception that foods derived from approved GE crops were toxic, and was followed by a series of studies, the majority of which originated in European laboratories, that reported adverse health effects of GE crops These studies have been reviewed and analyzed by Sánchez and Parrott (2017), who conclude that methodological flaws in these studies invalidate claims of adverse effects. The authors reaffirm the lack of good evidence for adverse health effects of any commercialized GE crop.

#### A series of papers published by Gilles-Eric Séralini and his laboratory

Among the most impactful of the subsequent papers were those published by the laboratory of Gilles-Eric Séralini. The first of these (Séralini et al. 2007) was a re-analysis of the safety data submitted by the agricultural biotechnology company, Monsanto, in support of its application for approval of maize event MON863 for food, feed and processing. Their re-analysis of the Monsanto data led them to conclude that the feeding studies the company conducted in fact indicated that the Bt maize caused several metabolic and clinical problems in the test animals. The European Food Safety Authority (EFSA) disagreed with the conclusions of the report (European Food Safety Authority 2007a, b) and therefore did not withdraw their approval of MON863. The Seralini group followed with a re-analysis of MON863 again, along with two other Bt maize events, MON810 and NK603, and came to similar conclusions about these three events as they did in the previous study (de Vendômois et al. 2009). EFSA reviewed the analysis conducted by de Vendômois et al. and decided that it did not present any new information regarding the toxicity of these three events (European Food Safety Authority 2010). A third publication reviewed data from the feeding studies of 19 approved GE crop events and concluded that these studies showed liver and kidney problems as well, and called for longer term feeding studies that included analysis of additional endpoints that were not required at the time (Séralini et al. 2011). This paper did not prompt EFSA to conduct a re-evaluation of the data from these studies.

Then in 2012, the Séralini group published the results of feeding studies conducted in their own laboratories, which showed that rats fed diets with maize event NK603 had higher death rates and developed more tumors than the control-fed rats (Séralini et al. 2012). The paper, and the manner in which the authors publicized it, was widely criticized by other scientists, (see for example Anonymous editor (2012)). The European Food Safety Authority was tasked with analyzing the Seralini et al. (2012) paper and found it to be “inadequately designed, analysed and reported”, and concluded that “the study as reported by Séralini et al. [was] of insufficient scientific quality for safety assessment” (European Food Safety Authority 2012). The paper was finally retracted by the journal, but then republished in another journal (Séralini et al. 2014). While no regulatory agency that had approved NK603 was prompted to change its conclusions as a result of the Séralini et al. paper, it nevertheless had a significant impact in at least one African country: Kenya. Because of this paper, in 2012 the Kenyan Minister of Public Health persuaded the country’s President to decree the removal of foods derived from GE crops from the market in Kenya and to ban imports of such foods. That ban remained in place until 2019 (Thomson 2021). Seralini’s group continued to publish papers showing health concerns with GE crops^43^, although those results have not been replicated by other laboratories.

The above examples of the impact that other scientists might have, whether intended or not, should be a cautionary tale for the field of gene drives. One example of a study that had similar but not as damaging an impact on the development of GE mosquitoes generally, was the publication of a paper by Evans et al. (2019), entitled “Transgenic *Aedes aegypti* Mosquitoes transfer Genes into a Natural Population”. The paper showed that some of the sterile males released by the company Oxitec to control mosquito-borne viral diseases had managed to hybridize with local populations, resulting in the introgression of parts of the release strain’s genome (but no transgenes) into those populations. This report resulted in concerns being raised in the press, with headlines such as “GM Mosquitoes Spreading Out of Control in Brazil”^44^. While the press article is consistent with the message that might be inferred from the title of the scientific publication, the data presented in it told a more sober story. Subsequently, an “expression of editorial concern” was published in the following year, which described problems that had come to the attention of the editors, regarding the interpretation of the data and some of the conclusions (Evans et al. 2020). Some of the authors disagreed with one of the conclusions of the paper that the hybrids between the released strain and the local populations “very likely result[ed] in a more robust population than the pre-release population due to hybrid vigor” (Evans et al. 2020), and some called for retraction of the paper^45^.

Of greater concern is that groups opposing the release of GE mosquitoes use extrapolations from the scientific literature to heighten concern about these organisms. An article contributed to the digital newspaper, *Huffpost*, by Jeffrey Smith entitled “Research Exposes New Health Risks of Genetically Modified Mosquitoes and Salmon”^46^, exemplifies the use of a common approach, which is to point to results of scientific studies showing biochemical or molecular differences between GE organisms and their non-GE counterparts, or to highlight a molecular phenomenon (in this case off-target effects of Cas9 in mice) and imply that these phenomena themselves are harmful effects and require further studies to show that they have not occurred. This article argues that the failure to do these studies are therefore an oversight in the safety evaluation and calls for a continued distrust of the GE organism until studies are conducted to show that these effects have not occurred. It should be noted that the scientific article referenced in the *HuffPost* article was not about mosquitoes nor salmon, yet those organisms are mentioned in the title. This approach was often used against GE crops, with success, and is now being used against GE mosquitoes. GE mosquitoes containing gene drives will most likely also be the subject of similar tactics.

Therefore, accurate information on gene drives, provided by established and recognized trustworthy sources, should be provided to the general public, journalists, and scientists who are not connected with the field. Those sources of information should also be able to summarize and analyze important scientific results and emerging issues in the field in order to place those developments in the proper context. Examples of such resources include organizations such as the GeneConveneVirtual Institute^47^, and the African Genetic Biocontrol Consortium^48^. Governments would also be the source of such trustworthy information (Sect. “Low public understanding of the technology”).

### Corporations as the first developers

For GE crops, resistance due to the distrust of regulators and science was heightened by the introduction of the first products by multinational companies. The tomato purée product was introduced by Zeneca, and other products that were soon to be introduced came from another multinational company, Monsanto, which did not conduct effective engagement with the public (Burkeman 1999; McCabe 1999; Reynolds 2004). The company was based in the United States, which brought with it negative connotations of industrial food production and fast-food culture (Fillipo Randazzo, personal communication). Being products of multinational corporations, and the lack of product counterparts in nature, contributed to the perception of these products as unnatural, and these perceptions carried over to subsequent products (Bruce 2017). Even today, the importance of having a counterpart or being derived from nature plays a deciding role in the acceptance of genetic engineering, with gene transfers between varieties of the same plant being possibly accepted by consumers in Europe (Saleh et al. 2021). Therefore, if those planning deployment of gene drives were to learn from the GE crop experience, the introduction should not be seen as being driven by companies, and the public should be made aware of natural gene drive counterparts. While this perception might seem straightforward to achieve, given that the developers of gene drive technology have thus far been not-for-profit research groups funded by private or government donors, it competes with a developing narrative that these initial efforts to deploy health benefits is a “smoke screen” for eventual agricultural applications by for-profit interests (ETC Group 2018; de Wit 2019). This narrative mirrors that of one of the criticisms of Golden Rice, a product developed by a consortium of six public institutions^49^, with extensive support from the Rockefeller Foundation, USAID, and the Bill and Melinda Gates Foundation (BMGF) (Potrykus 2001; Datta and Datta 2020)^50^. Groups opposing GE crop deployment argued that this pro-poor application of biotechnology was motivated by the desire to make GE crops more acceptable and therefore enhance the acceptance of other crops that would be the source of corporate profit (Enserink 2008; Fuchs and Glaab 2011; Kazumi 2020)^51^.

Furthermore, corporate ownership of the majority of GE crops made them vulnerable to criticisms of exerting undue control over the world food and seed supply. As GE crops became more successful and profitable, production consolidated to only a few companies: Monsanto, Syngenta, Dow AgroSciences, and DuPont/Pioneer, with Monsanto becoming the focus of much activist attention, even though they were not the largest member of the field^52^. This number has recently decreased further to Bayer, Dow AgroSciences, and Corteva^53^. The concentration of investments in GE crops is also a vulnerability of gene drives, which—while not being controlled by major commercial interests—is a field that is funded by only a few—primarily one—private donors. The significantly higher investment in gene drive research by BMGF than any other private donor has led to criticism from opposition groups and has been connected with suspicion to the major investment in gene drives by the US Defense Advanced Research Projects Agency^54^. The connection with multinational corporate giant Microsoft, whether that connection is real or not, will also continue to be associated with any charitable work supported by BMGF.

The research groups leading the efforts to develop and deploy gene drives in Africa are not-for-profit and university-based, funded by charitable donors. Thus, the resistance to corporate interests would seem to be overcome. However, the source of the current funding comes primarily from a few large donors, which makes the field susceptible to accusations of undue control by these donors. Therefore, diversifying the source of funding, including participation by countries, or regional bodies, would show that country or regional interests are represented by this work, and that the influence of large donors is diluted.

### Lack of identifiable consumer benefit

The first GE crop products also failed to have obvious benefits to the consumer. While the very first product (Flavr-Savr tomato) was clearly a consumer-oriented trait, it was distributed in an inferior tomato genotype^55^, and therefore did not provide a highly acceptable product, not only for the consumer but for the farmer as well. The tomato paste counterpart also did not provide obvious enhancement of consumer experience. The benefit of the tomato was reduced processing cost (Clark et al. 2004), and a lower price compared to non-GE tomato paste^56^. This combination apparently was not sufficiently compelling to the public (Sect. “Mistrust of government regulatory agencies”). Subsequent introduced products all expressed traits to benefit farmers—herbicide tolerance, insect and disease resistance—which drove their adoption worldwide (Sect. “Adoption of GE crops worldwide”). It remains to be seen whether Golden Rice, which has identifiable consumer benefit but has still not been commercially deployed anywhere in the world, presents a compelling enough story to counteract the already-entrenched opposition group narrative of GE crops as an example of corporate deception.

Fortunately for the gene drive development community, the benefit to the public (if it can be demonstrated convincingly) would be immediately obvious. However, this does not make gene drives free of criticisms that would place these benefits in a darker light. The monolithic donor narrative has already been mentioned. Furthermore, the potential effectiveness of the technology, possibly allowing suppression of disease vector populations that might not have been achievable by existing tools, has given rise to concerns about the eradication of mosquito species and the role of mosquitoes in the environment that were not expressed about other technologies (e.g., pesticides) deployed to achieve the same goal (National Academies of Sciences Engineering and Medicine 2016; Roberts et al. 2017; Collins et al. 2019; Teem et al. 2019; Connolly et al. 2021). Efforts by developers and more neutral groups (see Sect. “Scientific publications claiming adverse effects of GEOs” above) to provide a more thorough understanding of the benefits and risks of this technology would help the public and decisionmakers reach more informed decisions.

### Low public understanding of the technology

The subject of genetic engineering is a highly technical one, and is also highly charged, due to the polarized communication around this subject. Early studies on the public understanding of biotechnology, prior to or contemporary with the commercial deployment of transgenic crops, showed a lack of information about it, especially regarding food and agricultural biotechnology, resulting in a negative reaction to the technology (Harlander 1991; Frewer et al. 1994). The United States Office of Technology Assessment reported in 1987 that 69% of the American public surveyed had heard or read “almost nothing” or “relatively little” about genetic engineering. Seventy-five percent of the “almost nothing” category could not explain what genetic engineering was, and neither could almost 50% of the “relatively little” category (US Congress, OTA 1987). A Europe-wide survey of biotechnology attitudes, conducted in 1996, showed that only 36% of the public correctly identified as false the statement that “Ordinary tomatoes do not contain genes while genetically engineered tomatoes do.” (Melich 2000). Other surveys conducted by researchers in various countries worldwide at the same time corroborate these results (Davison et al. 1997). More recently, even after two decades since transgenic crops have been introduced, public understanding is still inadequate (Brossard 2018). The lack of widespread understanding exists even though the subject has had wide media coverage since the early days of the deployment of transgenic crops. This coverage has been viewed by scientists as being overly sensational, thus hindering accurate understanding; conversely, journalists viewed scientists as being unable to communicate effectively to the lay public (Gunter et al. 1999).

Countries in Africa have recognized the key role of communicating with the public about biotechnology, with some countries explicitly making this part of the role of their National Biosafety Authority, both as a means for public input, but also as a means of educating them. For example, the Kenyan biosafety law provides that the National Biosafety Authority “…promote awareness and education among the general public in matters relating to biosafety” (Government of Kenya 2009). Burkina Faso has similar language in its Biosafety Law: “L'Agence nationale de biosécurité, en collaboration avec les autres administrations concernées, veille à ce qu'il y ait une sensibilisation et le cas échéant, une consultation publique adéquate au sujet de l'utilisation, de la dissémination de tous les organismes génétiquement modifiés”^57^(Government of Burkina Faso 2012).

However, South Africa is the only country on the continent with a government program designed to promote a clear understanding of biotechnology. The Public Understanding of Biotechnology Program^58^ aims to present information on the subject clearly and fairly, in order to support an accurate understanding of the subject. Despite this effort, a recent survey in South Africa conducted to assess the effectiveness of the program, found that 73% of respondents reported having little or no knowledge about biotechnology and 46% viewed biotechnology as too specialized a field of knowledge for the public to understand (Gastrow et al. 2018).

Therefore, further efforts to enhance the understanding of genetic engineering and biotechnology are needed, in order to assure that accurate information about this technology is disseminated. A better understanding of gene drive technology can be fostered by governments, as part of established biosafety policy in several African countries. Developers and neutral groups could also be helpful in increasing public understanding of the technology of genetic engineering, including gene drives. Without this understanding as a base, it is likely that the understanding of gene drives will be even more difficult, resulting in the same negative reaction on the part of the public. While providing accurate information does not necessarily lead to acceptance or support, it will serve to balance an alarmist portrayal of genetic engineering on the part of groups who oppose it (see next section).

As the next section will explore, easily grasped messages and images can be effective ways to communicate about a technology and could be used by those who want to achieve acceptance of gene drives. Unfortunately, clear messages with emotional impact engendering fear and rejection of a technology are easier to achieve than similar messages that convey information about a complex subject, often delivered by scientists who are naturally careful to deliver accurate and non-misleading statements.

More accurate public understanding could benefit greatly from consistency in terminology used by researchers in the field when discussing the subject within their own community, but also by those in the field of gene drive when communicating with the public. One development along this line is an effort to standardize gene drive terminology among researchers in this field (Alphey et al. 2020). Another group is developing a glossary of terms translated into local languages in Burkina Faso, Mali, and Uganda (Chemonges Wanyama et al. 2021). At the very least, such consistency will avoid confusion when discussing the technology, and therefore encourage better understanding.

### Messaging to the public by opposition groups

#### Alternative vision of agriculture

Opponents of the use of GE crops have successfully leveraged perceptions of agriculture that view modern, industrial, high-intensity approaches as damaging to the environment and to society. These are agricultural practices that are employed to a great extent in many developed countries such as the United States, Canada, Argentina, and Brazil. These practices were, ironically, initiated by innovations of the Green Revolution aiming to maximize agricultural productivity in developing countries.

While considered one of the most significant technological advances of the twentieth century, resulting in the saving of over a billion lives, the Green Revolution has been the subject of criticism as well. Conway and Barbier (1990) have pointed out problems of equity (less suitable for low resource, small-holder farmers) and stability of production (less environmental resiliency of production due to monocropping). The Green Revolution has been viewed as advancing input-dependent, commercial farming (“global North” farming) instead of subsistence farming (Harwood 2019), and the cause of ecological damage and violence in the Punjab region of India (Shiva 1991). Therefore, industrialized farming was regarded by some as a negative outcome of agricultural technological advancement sparked by the Green Revolution, notwithstanding the increased economic and health improvements it produced.

The industrialized farming encouraged by the Green Revolution is viewed by some as inherently unsustainable and damaging to the environment (Clunies-Ross and Hildyard 2013). This view is also espoused by the agroecological movement, which includes practices to intentionally preserve biodiversity in order to take advantage of the ecosystem services that it provides. It stands as an alternative to industrial agriculture (Kremen et al. 2012). As discussed in the “Regulatory status in Europe” section, the agroecological movement is highly influential in European agricultural policy.

Since GE crops are associated with industrial agriculture, the connotations (whether real or perceived) that go along with that association have therefore resulted in GE crops being viewed as advancing an anti-biodiversity, non-sustainable way of agriculture. This perception persists despite the data to the contrary, as described in the “Benefits of adoption of these crops” section. The introduction of GE crops has been seen as a continuation of technological dependence and factory farming^59^. Thus, efforts to expand Green Revolution practices to Africa by humanitarian organizations such as the Bill and Melinda Gates Foundation have been criticized for encouraging policies that are inappropriate for the continent and exerting undue influence on academic institutions to achieve their goals^60^. The foundation has also come under criticism for being institutionally unsuited to listen to smallholder farmer concerns, despite a genuine desire to serve them (Schurman 2018). The reaction against industrialized farming is especially intense in Europe, and GE crops have become a focal point for these two approaches and the values they represent (Levidow and Boschert 2008), as discussed in the “Opposition to GE crops in Europe and its impact on Africa” section.

The opposition to gene drives appears to be developing along similar lines. There has been criticism that the use of gene drives arises from the same mentality as that which gave rise to the Green Revolution:

“The same mindset, which led to the stockpiling of chemicals of war in our fields with the Green Revolution, later developed today’s failed genetically engineered herbicide resistant crops… Monsanto & Co – which includes investors, scientists, corporations, DARPA, and Gates Foundation – continues doggedly to rely on this misguided ‘techno-fix’ approach, now with gene drives technology to solve the failures they have created themselves, another tool on the path of unbridled profit and control.”

--Navdanya International^61^

Gene drives have been positioned as unsustainable technological solutions that will not succeed, as opposed to less technologically reliant solutions that will. Regarding the use of gene drives for conservation purposes, the following view has been expressed:

“Genetic extinction technologies are a false and dangerous solution to the problem of biodiversity loss. There are real, sustainable, community-based conservation efforts that should be supported.”

--Erich Pica, President, Friends of the Earth^62^

Therefore, in delivering messages about gene drives, it will be necessary to avoid the perception that this technology is a developed country technology that is being inappropriately applied to developing countries. Co-development with local scientists, could be important in developing ownership of the technology, much as it has done with recent GE crop introductions^63^^,^^64^. Participatory modeling to engage communities in the decision-making and planning process, described in “Mistrust of government regulatory agencies” section, could also ensure the appropriate deployment of this technology.

#### Fear and uncertainty associated with new technology

**Fig. 2:**
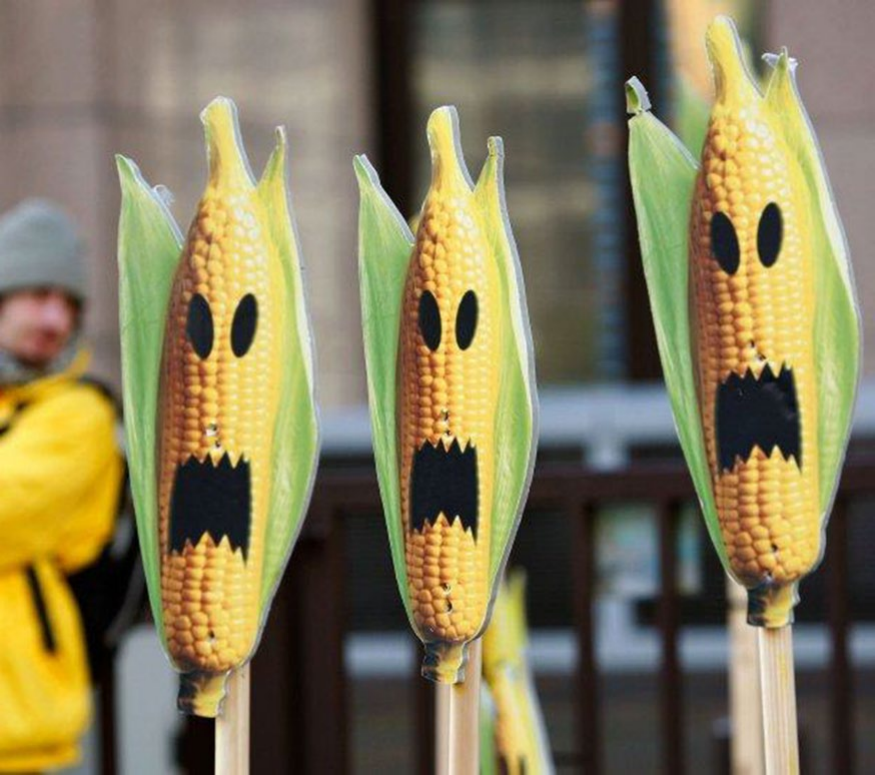
Depiction of GE maize by Greenpeace (© Reuters, used with permission)

Within the context of opposition to agricultural technology, visually powerful images are well used by opposition groups to inspire fear of the technology. An example of such images is provided in Fig. 2. These messages take advantage of a well-established “negativity bias” that exists in framing impressions of individuals as well as establishing attitudes (see Fridkin and Kenney 2008). Thus, it is more difficult to gain acceptance of an idea via positive messaging. Groups that are associated with environmental protection also tend to be more trusted than the government (See US Congress, OTA 1987 above).

Another messaging strategy has been mentioned previously: pointing out differences between GEOs and their counterparts or pointing to issues of scientific concern or uncertainty and citing those issues as evidence of harm. As Juma (2016) states, “The potential of harm (hazard) was over time presented to the general public as probability of the harm occurring.” An example of the use of this strategy with respect to GE insects (and probably GE insects containing gene drives) was noted previously. This type of messaging reinforces the view that GE technology is inherently unsafe, which has resonated powerfully with the public.

This messaging about GE organisms has enabled opposition groups, dependent on charitable donor funding, to establish an “economic engine” to fund their work. The strong monetary support totaled almost $1 billion in the period 2012–2016^65^, funding the efforts to oppose the development of GE technology worldwide. The extent of these efforts is out of scope for this report but are thoroughly described by Jennifer Thomson in Chapters 9 and 10 of her recent book, *GE Crops and the Global Divide* (Thomson 2021). Presently, efforts of these groups remain focused on GE technology in agriculture, but there is growing involvement of these same entities into GE insects. For example, the Center for Food Safety and Organic Consumers Association was active in opposing Oxitec’s work with mosquitoes in the Florida Keys^66^.

While these messages seem to have been effective in driving opposition to GE crops, there could be an opportunity to shape public perception regarding gene drives, because this area of genetic engineering is still relatively new. A study conducted in New Zealand (MacDonald et al. 2020; 2021) found that opinions about gene drives have not become entrenched in that country, leading the authors to recommend early and effective engagement with the public if support for the technology is desired by developers and funders. The same might be true for other countries or regions of the world, although public opinion might already be solidifying against the technology in Europe (Sect. “Developing opposition to gene drives in Europe”). Therefore, if gene drive technology proves to be a beneficial tool for the elimination of malaria or other diseases, better messaging about these benefits, as counterpoints to messages coming from groups opposed to the technology, would enable a more balanced public view of the technology. These messages should strive to have the same type of emotional and intuitive appeal as those that have been developed in opposition. For example, the power of personal experiences and stories are more effective than delivery of factual information in certain cases (Freling et al. 2020; Kubin et al. 2021).

## Concluding remarks

The technical success of GE crops has not been matched by success in the public acceptance of the technology on the part of the general public, particularly in Africa. This disconnect has been the result of several factors, many of which have been reviewed in this report. There have been errors made by the developer community, particularly in their engagement with the public. On the other hand, there have been successes on the part of groups that oppose the development of GE technology, taking advantage of their connection with that same public and the inherent communication advantages that such groups enjoy. There have also been historical events that have placed GE crops in a difficult receiving environment. The factors can serve as lessons learned, some of which can be the motivation for adopting policies and strategies that would avoid a similar effect on gene drive development. Some of these, such as the lack of accurate public understanding of the technology or the lack of effective messaging to the general public are already being experienced by the gene drive development community, which is working to overcome them. However, in addition to providing accurate technical information about gene drive technology, providing an understanding of its value—along with concomitant risks and uncertainties—should be an overarching goal. Facts alone will not serve to convince the public of any course of action, or to even weigh alternatives fairly. Other factors, such as the influence of European attitudes on African policies, or the publication of work that inadvertently or intentionally raises public concerns about the safety of gene drives, do not lend themselves to a solution, but nevertheless are items to recognize, so that mitigation measures can be developed to minimize the damage that might occur.

## Footnotes

1 https://www.une.edu/news/2019/thomas-klaks-project-restore-american-chestnut-tree-featured-news-center-maines-207.

2 https://www.ers.usda.gov/webdocs/DataFiles/47649/alltables.xls?v=6605.8.

3 https://www.ers.usda.gov/data-products/adoption-of-genetically-engineered-crops-in-the-us/recent-trends-in-ge-adoption.

4 https://supportprecisionagriculture.org/nobel-laureate-gmo-letter_rjr.html.

5 see for example, https://www.canr.msu.edu/news/are-gmos-safe.

6 This does not imply that there is no opposition in other countries. Analyses of attitudes has toward GE crops been conducted in recent years for the United States (Hasell et al. 2020), China (Xu et al. 2020), and India (Navneet 2020). Interestingly, in the light of GE organisms containing gene drives, GE crops and other “traditional” transgenic products recently seem to be viewed in a more benign light, due to their familiarity, even by long-term opponents of GEOs (Dolezel et al. 2020).

7 https://www.conseil-etat.fr/ressources/decisions-contentieuses/dernieres-decisions-importantes/conseil-d-etat-7-fevrier-2020-organismes-obtenus-par-mutagenese.

8 https://ec.europa.eu/food/plants/genetically-modified-organisms/gmo-authorisation_en.

9 Approvals of GE products are issued for “events”, i.e. an initial molecular insertion into a plant and subsequent offspring derived from sexual reproduction or asexual propagation.

10 http://www.isaaa.org/gmapprovaldatabase/advsearch/default.asp?CropID=Any&TraitTypeID=Any&DeveloperID=Any&CountryID=EU&ApprovalTypeID=3.

11 When it was a member of the European Union, the United Kingdom was generally aligned with this more precautionary approach. However, since that country’s departure from the EU, there could be a change in its regulatory attitude. A report published by the UK’s Regulatory Horizons Council (Leurs 2021) shows a willingness to re-examine the regulatory approach to genetic technologies, focused on agricultural applications.

12 Although cultivation of GE crops is limited in the EU, there are over 50 GE plant events that are authorized in the EU for food and feed import and processing (https://ec.europa.eu/food/plant/gmo/eu_register_en; https://webgate.ec.europa.eu/dyna/gm_register/index_en.cfm). In fact, animal feed in Europe is highly dependent upon the import of these GEOs (https://tillymetz.lu/wp-content/uploads/2020/09/Co-signed-letter-MEP-Metz.pdf). Therefore, there are products in Europe that contain GE components, and it would be of interest to determine whether the European consumer deliberately avoids these products, or is unaware that that they might be consuming such products if they do not reach the threshold for labeling of 0.9% (https://eur-lex.europa.eu/legal-content/EN/TXT/?uri=celex:32003R1830).

13 The most commonly used, *npt2*, has been judged safe by the European Food Safety Authority, https://efsa.onlinelibrary.wiley.com/doi/abs/10.2903/j.efsa.2007.742

14 See https://ec.europa.eu/food/plant/gmo/authorisation/decision_making_process_en for a graphical representation.

15 https://www.soilassociation.org/causes-campaigns/a-ten-year-transition-to-agroecology/what-is-agroecology.

16 https://www.organicseurope.bio/what-we-do/cap-and-rural-development/.

17 https://ec.europa.eu/info/strategy/priorities-2019-2024/european-green-deal_en.

18 http://www.fao.org/english/newsroom/news/2002/8660-en.html.

19 https://documents.wfp.org/stellent/groups/public/documents/newsroom/wfp076534.pdf.

20 https://www.theguardian.com/science/2002/oct/07/gm.famine.

21 https://dailynews.co.zw/govt-lifts-ban-on-gmo-maize/https://www.newzimbabwe.com/starving-zim-reconsiders-producing-genetically-modified-crops.

22 https://www.newzimbabwe.com/starving-zim-reconsiders-producing-genetically-modified-crops/.

23 https://www.isaaa.org/kc/cropbiotechupdate/article/default.asp?ID=18716.

24 https://www.pollinis.org/admin/wp-content/uploads/2021/01/resultats-sondage-forcage-genetique.pdf.

25 see for example https://www.stop-genedrives.eu.

26 https://www.europarl.europa.eu/doceo/document/TA-9-2020-0015_EN.html.

27 https://www.stop-genedrives.eu/en/european-parliament-calls-for-a-global-gene-drive-moratorium/

28 https://au.int/sites/default/files/decisions/33909-ex_cl_decisions_986-1007_e.pdf.

29 https://www.nepad.org/news/west-africa-integrated-vector-management-steering-committee-adopted-workplan-and.

30 https://www.euractiv.com/section/health-consumers/opinion/how-brussels-can-help-or-hinder-the-fight-against-malaria/.

31 https://api.parliament.uk/historic-hansard/commons/1996/mar/20/bse-health.

32 One recent informal follow-up of the Okorie et al. work can be found at https://thoughtleader.co.za/do-africans-need-genetically-modified-mosquitoes/.

33 https://nbma.gov.ng.

34 https://grain.org/article/entries/427-twelve-reasons-for-africa-to-reject-gm-crops.

35 GMO is synonymous with the term “GE organism used in this paper.

36 https://sustainablepulse.com/2015/10/22/gm-crops-now-banned-in-36-countries-worldwide-sustainable-pulse-research/.

37 https://www.uncst.go.ug/national-biosafety-committee/.

38 https://geneticliteracyproject.org/2020/07/13/uganda-proposes-stop-gap-rules-to-regulate-and-block-gmo-crops-that-could-be-brought-illegal-across-the-kenyan-border/.

39 https://www.motherjones.com/food/2012/03/researchers-gm-crops-are-killing-monarch-butterflies-after-all.

40 https://www.gmoevidence.com/cornell-university-bt-corn-harms-monarch-butterfly-larvae/.

41 see for example https://www.thelancet.com/journals/lancet/article/PIIS0140-6736(99)90199-X/fulltext.

42 see for example https://www.independent.co.uk/climate-change/news/scientists-revolt-at-publication-of-flawed-gm-study-737888.html.

43 https://www.gmoseralini.org/research-papers.

44 https://sustainablepulse.com/2019/09/11/gm-mosquitoes-spreading-out-of-control-in-brazil-as-genome-found-in-wild-populations/.

45 https://www.sciencemag.org/news/2019/10/dissent-splits-authors-provocative-transgenic-mosquito-study.

46 https://www.huffpost.com/entry/research-exposes-new-health-risks-of-genetically-modified_b_597a3cb4e4b06b305561cef3.

47 https://www.geneconvenevi.org/

48 https://www.genbioconsortium.africa/

49 the Bangladesh Agricultural University (BAU), the Bangladesh Rice Research Institute (BRRI), the Donald Danforth Plant Science Center in the USA, the International Rice Research Institute (IRRI), the Philippine Rice Research Institute (PhilRice), and the University of Freiburg in Germany http://goldenrice.org/index.php

50 see also https://gcgh.grandchallenges.org/challenge/create-full-range-optimal-bioavailable-nutrients-single-staple-plant-species.

51 see also https://www.organicconsumers.org/news/monsantobill-gates-plot-genetically-engineered-rice-threatens-asian-countries and https://www.independentsciencenews.org/health/why-we-oppose-golden-rice/?utm_source=rss&utm_medium=rss&utm_campaign=why-we-oppose-golden-rice.

52 see for example https://www.organicconsumers.org/campaigns/millions-against-monsanto.

53 https://www.gmwatch.org/en/news/latest-news/17328-dow-and-dupont-bayer-and-monsanto-or-syngenta-and-chemchina-which-will-succeed, https://www.reuters.com/article/us-monsanto-m-a-bayer-deal-idUSKCN11K128, https://www.dupont.com/news/dowdupont-agriculture-division-to-become-corteva-agriscience.htm.

54 https://stillnessinthestorm.com/2020/06/bill-gates-foundation-working-with-darpa-on-gene-editing/, https://foe.org/news/military-revealed-top-funder-gene-drives-gates-foundation-paid-1-6-million-influence-un-gene-drives/.

55 https://www.bionity.com/en/encyclopedia/Flavr_Savr.html.

56 https://beyond-gm.org/20-years-ago-today-what-have-we-learned-since-the-gmo-flavr-savr-tomato/.

57 Translation: “The National Biosecurity Agency, in cooperation with the other involved administrations, ensures that sensitization occurs and, if necessary, an adequate public consultation on the use and release of all genetically modified organisms.”

58 https://www.pub.ac.za.

59 https://beyond-gm.org/20-years-ago-today-what-have-we-learned-since-the-gmo-flavr-savr-tomato/.

60 https://cagj.org/wp-content/uploads/Messengers-of-Gates%E2%80%99-Agenda-A-Case-Study-of-the-Cornell-Alliance-for-Science-Global-Leadership-Fellows-Program.pdf.

61 https://navdanyainternational.org/gene-drive-extinction-technology/#:~:text=Gene%20drives%20technology%20is%20a%20rough%20tool%2C%20based,organisms%20as%20well%20as%20their%20complex%2C%20dynamic%20evolution.

62 https://foe.org/news/2016-08-genetic-extinction-technology-rejected-by-international-group-of-scientists/.

63 https://www.ofabnigeria.com/2022/01/12/nigeria-launches-bt-cowpea-commences-commercial-production/.

64 https://africa.cgtn.com/2021/10/29/kenya-set-to-launch-genetically-modified-cassava/.

65 https://anti-gmo-advocacy-funding-tracker.geneticliteracyproject.org.

66 http://kfolta.blogspot.com/2021/04/consumer-advocates-or-anti-biotech-in.html.
